# A novel route to product specificity in the Suv4-20 family of histone H4K20 methyltransferases

**DOI:** 10.1093/nar/gkt776

**Published:** 2013-09-18

**Authors:** Stacey M. Southall, Nora B. Cronin, Jon R. Wilson

**Affiliations:** Division of Structural Biology, Institute of Cancer Research, Chester Beatty Laboratories, London, SW3 6JB, UK

## Abstract

The delivery of site-specific post-translational modifications to histones generates an epigenetic regulatory network that directs fundamental DNA-mediated processes and governs key stages in development. Methylation of histone H4 lysine-20 has been implicated in DNA repair, transcriptional silencing, genomic stability and regulation of replication. We present the structure of the histone H4K20 methyltransferase Suv4-20h2 in complex with its histone H4 peptide substrate and S-adenosyl methionine cofactor. Analysis of the structure reveals that the Suv4-20h2 active site diverges from the canonical SET domain configuration and generates a high degree of both substrate and product specificity. Together with supporting biochemical data comparing Suv4-20h1 and Suv4-20h2, we demonstrate that the Suv4-20 family enzymes take a previously mono-methylated H4K20 substrate and generate an exclusively di-methylated product. We therefore predict that other enzymes are responsible for the tri-methylation of histone H4K20 that marks silenced heterochromatin.

## INTRODUCTION

Histone lysine methyltransferases generate site-specific modifications in chromatin that signal fundamental events in transcription, replication and DNA repair (1,2). Lysine methylation has particular potency in chromatin signalling as each lysine can be mono-, di- or tri- methylated to create discrete binding sites. In turn, effector proteins contain recognition domains that not only exhibit a high degree of sequence specificity but also are sensitive to the level of methylation (3,4). Only a limited number of histone lysine side-chains have been implicated in epigenetic signalling, and for histone H4 in higher organisms, only the lysine-20 residue is methylated. In *Schizosaccharomyces pombe*, all histone H4K20 methylation has been attributed to a single SET domain methyltransferase, Set9 (5). However, in higher organisms, multiple enzymes have evolved to control histone H4K20 methylation; established H4K20-specific methyltransferases include PR-Set7 and the two Set9 orthologs Suv4-20h1 and Suv4-20h2, meanwhile histone H4 lysine-20 monomethyl mark (H4K20me1) demethylase activity has been demonstrated for PHF8 (6–9).

A fundamental concept that underpins epigenetic signalling is that each modification state should lead to a specific biological outcome. In the case of H4K20 methylation, a range of studies have linked H4K20me1 with the regulation of mitotic regulation and the timing of replication (10–12) and H4K20me3 with transcriptional silencing (9,13–15). However, analysis of chromatin isolated from *Drosophila* S2 cells and a human *HeLa* S3 cell line using mass spectrometry revealed that globally as much as 90% of histone H4 is di-methylated at lysine-20, making it by far the most prevalent mark (16,17). Can such a ubiquitous mark have a regulatory role? Direct and preferential binding of H4K20me2 by 53BP1 links this modification to signalling in double-strand DNA break repair (5,18,19). Analysis of replication origins using ChIP assay showed an increased H4K20me2 signal, which correlated with recruitment of the Orc1 protein through its the BAH domain (20). This recruitment is required for DNA replication licencing and cell cycle progression. The divergent biological roles linked to the different H4K20 methylation states argue for the regulated maintenance of this mark (21).

Structural, biochemical and cellular studies have established that PR-Set7 activity is limited to H4K20 monomethylation (10,22,23). However, the knockout of PR-Set7 in mice leads to a global loss of all three methylated forms of H4K20. This implies that in higher organisms the PR-Set7 enzyme generates H4K20me1, and then subsequently other enzymes generate the H4K20me2 and H4K20me3 states. In higher eukaryotes, the Suv4-20 proteins have been shown to specifically methylate histone H4K20 *in vivo* (9,17,24,25). Whereas lower eukaryotes have a single Suv4-20 ortholog, mammals have two closely related Suv4-20 paralogs, Suv4-20h1 and Suv4-20h2. A strictly sequential H4K20 methylation model, in which Suv4-20 enzymes provide the next level of methylation after PR-Set7, was supported by a study in *Drosophila* that used RNAi silencing to reduce levels of the single Suv4-20 paralog—a decrease in the levels of both H4K20me2 and H4K20me3 was observed along with the accumulation of H4K20me1 (17). This pattern was replicated in similar experiments that have targeted Suv4-20 protein expression from fish to mammals (13,20,26). Suv4-20h1 and Suv4-20h2 have been shown to preferentially methylate histone H4K20 on nucleosomes (9), and when overexpressed in HeLa cells, both proteins localized to pericentric heterochromatin (27). However, in mice, although ubiquitous *Suv4-20h1* expression was observed in both embryo and adult tissue, *Suv4-20h2* expression was low in the embryo and limited to only a few adult tissue types (26). In the same study, *Suv4-20h1^−^^/^^−^* mice suffered from developmental defects that lead to perinatal lethality, whereas *Suv4-20h2^−^^/^^−^* mice developed normally. Knockout of Suv4-20h2 but not Suv4-20h1 in mouse embryonic fibroblast cells resulted in a reduction of H4K20me3 at pericentric chromatin and telomeres, with concomitant deregulation of telomere length (13,26). The accumulating evidence therefore argues that Suv4-20h1 and Suv4-20h2 have non-redundant roles.

**Figure 1.**
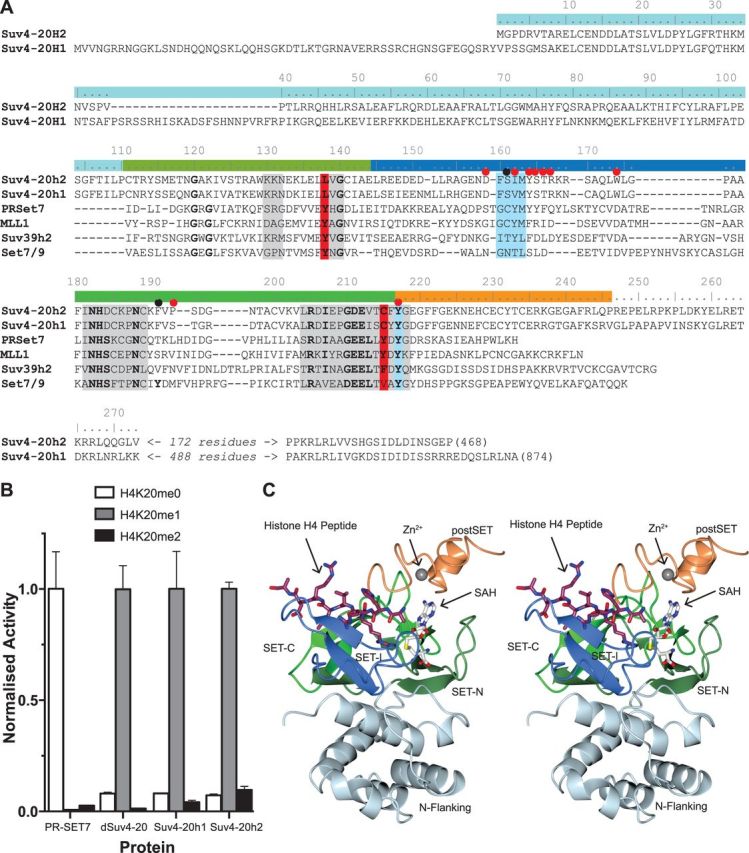
Intrinsic properties of the Suv4-20 family of SET domain methyltransferases. (**A**) Sequence alignment of Suv4-20 enzymes with other structurally characterized SET domains. The numbering is for Suv4-20h2, and the coloured bars indicate the sequence included in the structure construct and region of SET domain as indicated in (**C**). Conserved regions are indicated by a grey background. The residues involved in formation of the lysine channel in blue. Red circles indicate the residues involved in determining the specificity of Suv4-20h2. (**B**) Intrinsic methylation specificity of H4K20 specific methyltransferases. Recombinant PR-Set7, mouse Suv4-20h1 and Suv4-20h2 and *Drosophila* Suv4-20 activity was measured against peptide substrates based on the H4 sequence with different methylation states at the lysine 20 position. Activities were normalized to the most active substrate. (C) Stereo representation of the structure of Suv4-20h2 in complex with SAH and histone H4K20me2 peptide—the subdomains are coloured as indicated in the sequence alignment.

The methylation states of a defined subset of histone lysine residues determine the site-specific recruitment of effector proteins, and it is therefore essential that modification enzymes exhibit a high degree of product specificity. The SET domain protein lysine methyltransferase family is responsible for generating different site and methylation state specific lysine methylation in histone tails (28,29). The methylation state specificity in this family is determined by a well-documented mechanism termed the phenylalanine–tyrosine switch (23,30,31). However, a striking feature arising from sequence comparison of the Suv4-20 proteins with other SET domains of known structure is that the two aromatic residues that interact with the target lysine Nε and are the key component of this mechanism are not conserved (Figure 1A). We have determined the structure of the ternary complex of Suv4-20h2 with cofactor and histone H4 peptide and present details of a novel mechanism that accounts for both substrate and product specificity in the Suv4-20 enzyme family.

## MATERIALS AND METHODS

### Protein expression and purification

Murine Suv4-20h1(61-351), Suv4-20h1(61-327), Suv4-20h2(1-268) and *Drosophila* Suv4-20(147-393) SET domain constructs were expressed as Glutathione S-Transferase (GST)-fusion proteins. Suv4-20h2(1-246), used for crystallization was expressed with an N-terminal 6xHis tag. Mutations were generated using the Quikchange PCR mutagenesis method (Agilent). All constructs were expressed in *Escherichia coli* BL21 RIL cells (Agilent). GST-fusion proteins were isolated from clarified lysates using glutathione sepharose affinity resin (GE Healthcare) and separated from the tag by cleavage with rhinovirus 3C protease. His-tagged proteins were isolated on HisTrap FF columns (GE Healthcare), eluted with imidazole, and the tag was removed using Tobacco Etch Virus (TEV) protease. The protein was further purified by ion-exchange chromatography using HiTrapQ HP columns (GE Healthcare). All proteins were finally purified by size-exclusion chromatography (Superdex S75, GE Healthcare). The purification buffer was 20 mM HEPES (pH 8.0), 500 mM NaCl, 5 mM 2-mercaptoethanol (2-ME) (pH 8.0).

### Crystallization

Crystals were obtained by the hanging-drop method. A solution of mouse Suv4-20h2(1-246) (175 µM) with S-adenosyl homocysteine (SAH) (600 µM) and a histone H4 peptide (AKRHRK(me2)VLRD-Y) (600µM), was crystallized in a condition consisting of 0.1 M BisTris (pH 6.5) and PEG 3350 (6–10%) and improved through rounds of seeding. Crystals were harvested into a cryobuffer consisting of the reservoir condition with 20% ethylene glycol and flash-frozen in liquid nitrogen. The Suv4-20h1(61-327) crystals were grown in a condition containing 0.1 M HEPES (pH 8.0), 10% PEG 10 000, improved with seeding and harvested into a cryo-buffer supplemented with 25% glycerol.

### Structure determination

Data for the Suv4-20h2(1-246) ternary complex and Suv4-20h1(61-327) binary complex were collected at the Diamond Light Source (Diamond Light Source Ltd, Harwell Science and Innovation Campus, Oxfordshire, UK) on station I02 and IO4-1, respectively. The reflections were indexed using XDS (32) and reduced/scaled with programs from the CCP4i suite (33). The Suv4-20h2(1-246) structure was solved by molecular replacement using the PHASER package (34) using the coordinates of human Suv4-20H2 from the binary complex with S-adenosyl methionine (SAM) deposited by the Structural Genomic Consortium (35) as the search model, PDB code 3RQ4 (36). Difference maps were used to rebuild and extend the initial model using the Coot molecular graphics package (37). Iterative cycles of refinement were carried out using REFMAC (38). The C-terminus of molecule B was poorly ordered, and all analysis presented refers to molecule A. The data for Suv4-20h1(61-327) were indexed with the iMOSFLM package (39) and then solved by molecular replacement using the PHASER package (34) with the mouse Suv4-20h2 structure as the starting model. The coordinates and structure factors for both structures have been deposited at the PDB with accession codes 4AU7 and 4BUP, respectively.

### Methyltransferase assays

Methyltransferase assays were performed using peptide substrates based on the histone H4 amino terminal sequence (KGGAKRHRKVLRDNIQ-Y) (Pepceuticals) either unmodified, mono- or di-methylated in the underlined position. Two assay formats were used. (i) An end-point assay that measured the incorporation of ^3^H labeled SAM into the peptide, which depends on the separation of the peptide from cofactor using C18 cartridge purification (Waters), previously described in (40). Final reagent concentrations were 0.5 mM peptide, 0.5 mM SAM (including 0.625 μM ^3^H SAM), in an assay buffer of 20 mM HEPES (pH 8.0), 50 mM NaCl, 2 mM 2-ME (pH 8.0). Assays were carried out at 22°C for 20 min with a final enzyme concentration of 3 μM and raw Disintegrations Per Minute (DPM) scintillation counter data converted to nmoles CH3/min/μmol, assuming 1 DPM is equivalent to 5.7 × 10^−^^15^ mmoles CH_3_. This conversion assumes a radiolabel stock chemical concentration of 12.5 μM and that there is complete recovery of labeled peptide. All assays were carried out in triplicate and expressed as mean ± standard deviation. Assays to obtain kinetic parameters were carried out as mentioned previously but using peptide concentrations in the range from 0 to 1.5 mM. Kinetic analysis of reaction rates was performed using the GraphPad Prism software package (GraphPad Software, Inc.). (ii) A continuous assay system, exploiting a coupled enzyme system that measures the generation of SAH using the Methyltransferase Colorimetric Kit (Cayman Chemical Company, USA), was used to follow the time course of the reaction. The manufacturer’s protocol was modified as follows: the final SAM concentration was adjusted to 400 μM, final peptide 200 μM and final enzyme concentration 2 μM. Detection of the final H_2_O_2_ product of the coupled enzyme series by 3,5-dichloro-2-hydroxybenzenesulfonic acid was measured at 515 nm at 1 min intervals for 150 min at 30°C. Measurements were performed in triplicate, the average reading at 10 min intervals were plotted with the standard deviation.

### Isothermal titration calorimetry

Isothermal titration calorimetry measurements were performed at 20°C, using an ITC 200 microcalorimeter (MicroCal Inc.). Determinations of the affinity for SAM were performed by injecting SAM into a sample cell containing Suv4-20h2 (1-268) or Suv4-20h1 (61-351) in 40 mM HEPES (pH 7.5), 500 mM NaCl and 2 mM 2-ME. Binding isotherms were analysed using Origin Software (OriginLab Corporation).

### Microscale thermophoresis

Binding measurements by microscale thermophoresis (MST) were performed using a NanoTemper Monoloth NT.115 Instrument (NanoTemper Technologies GmbH). A peptide corresponding to histone H4K20me1 (KRHRK(me1)VLRD) was synthesised with an N-terminal fluorescein tag (Pepceuticals) for MST measurements. Measurements were made at 20°C using 25% light-emitting diode power and 40% infrared-laser power with laser-on time was 30 s and laser-off time 5 s. Multiple measurements were made for each protein.

## RESULTS

### Intrinsic substrate and product specificity of the Suv4-20 family enzymes

Our initial aim was to establish the intrinsic methylation state specificity of the Suv4-20 family enzymes, by measuring the methyltransferase activity of recombinant constructs containing the SET domain with synthetic histone H4K20 peptide substrates (Figure 1B). Whereas PR-Set7 only displays activity with an unmodified substrate, consistent with it being a monomethylase, *Drosophila* Suv4-20 and both mouse Suv4-20h1 and Suv4-20h2 only show appreciable activity with a peptide that is monomethylated at H4K20. We conclude that the Suv4-20 family enzymes require a histone H4K20me1 modified substrate and then add only a single methyl group to produce a final product of H4K20me2. This supports the sequential model of H4K20 methylation but moreover implies that a different enzyme generates H4K20me3.

To determine the molecular basis of Suv4-20 specificity, we obtained crystals of a ternary complex of mouse Suv4-20h2 (residues 1–246) with the cofactor product SAH and a short peptide based on the histone H4 sequence and di-methylated at the K20 position—the product complex. A structure with a resolution of 2.1 Å was solved using a molecular replacement model of the structure of the human Suv4-20H2 binary complex with SAM (36). The overall structure is presented in Figure 1C, and the crystallographic statistics is in Table 1. When compared with other characterized SET domain proteins, key sequence features, such as the conserved active site tyrosine residues, are missing (Figure 1A, red columns). However, the characteristic SET domain topology is retained in the Suv4-20h2 structure (Figure 1C). Significantly, the SAH cofactor binds in a surface pocket on one side of the protein, and the main chain of the substrate peptide is located in a groove formed by the packing of the SET-I and postSET regions. Suv4-20h2 has a distinctive N-flanking domain that forms a four-helix bundle that comprises the entire region N-terminal to the SET domain, with one pair of these helices packing against the base of the SET domain.

**Table 1.** Crystallographic statistics, collection and refinement

Protein data bank Code	Suv4-20h2 ternary complex (4AU7)	Suv4-20h1 binary complex (4BUP)
Data collection		
Space group	P2_1_2_1_2_1_	P2_1_
Cell Dimensions		
a, b, c (Å)	37.3, 65.2, 209.5	46.3, 50.0, 129.4
α, β, γ (°)	90.0, 90.0, 90.0	90.0, 92.8, 90.0
Resolution (Å)	55.3-2.1 (2.13–2.07)	50.0-2.2 (2.28–2.17)
R_merge_	0.036 (0.36)^a^	0.09 (0.40)
Mn I/σI	16.9 (2.0)^a^	13.1 (4.3)
Completeness (%)	97.3 (82.0)^a^	91.4 (90.2)
Multiplicity	3.4 (2.1)^a^	6.2 (6.0)
Refinement		
Resolution (Å)	2.1 Å	2.2 Å
No. of reflections	25 953	28 977
R_work_^b^/R_free_^c^	0.19/0.24	0.20/0.26
No. of Atoms		
Protein	3677	3995
Ligand/Ion	29	8
Solvent	211	198
B-factors		
Protein	32.9	32.0
Ligand/Ion	23.9	29.8
Solvent	44.8	33.1
R.m.s deviations		
Bond lengths (Å)	0.017	0.012
Bond angles	1.88°	1.15°

^a^The average value across the resolution range, whereas that in parentheses is the value for the highest resolution bin.

^b^R_work_ = Σ | |F_o_| - |F_c_| |/Σ |F_o_|.

^c^R_free_ = Σ_T_ | |F_o_| - |F_c_| |/Σ_T_ |F_o_|, where T is a test data set of 5% of the total reflections randomly chosen and set aside before refinement.

### Suv4-20h2 specificity is generated by a novel active site configuration

**Figure 2.**
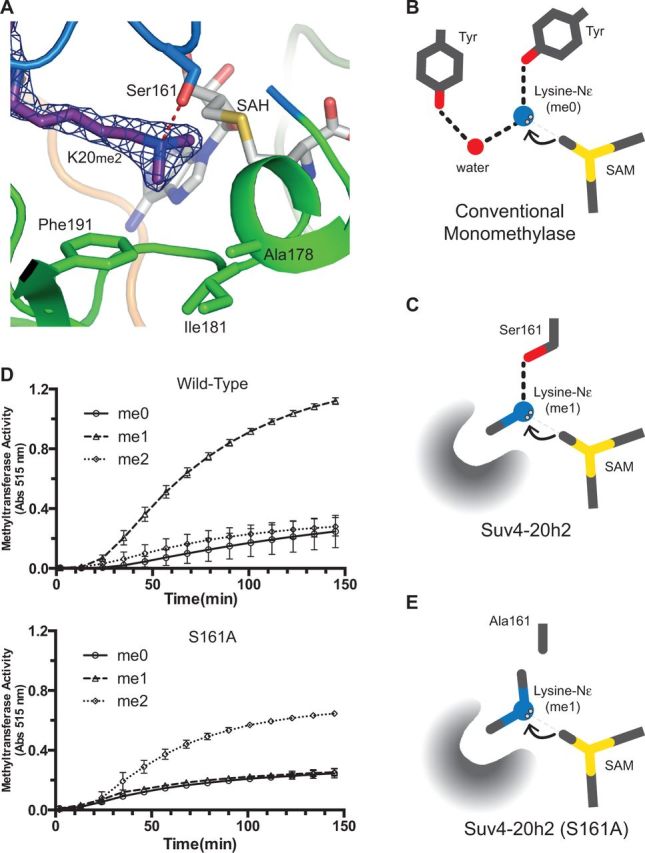
Mechanism of specificity. (**A**) The Suv4-20h2 active site showing the electron density for the substrate lysine side chain and methyls. Colours are as described for Figure 1C. (**B**) Schematic diagram showing the characteristic features of a canonical SET domain monomethylase. (**C**) Schematic diagram showing the features that define specificity in Suv4-20h2. (**D**) Activity of the wild-type and Ser161 to Ala mutant of Suv4-20h2. (**E**) A speculative schematic diagram illustrating the proposed configuration of a Suv4-20h2(S161A) complex.

The methyltransferase mechanism of the SET domain depends on the formation of a binding channel to restrain the target lysine in a position optimal for methyl transfer (28). For Suv4-20h2, this archetypal configuration is conserved, and the aliphatic portion of the target lysine side chain is constrained by aromatic side chains (principally Tyr217) and a main-chain tetrapeptide consisting of residues Phe160 to Met163. However, features that are unique to the Suv4-20 family of enzymes explain the molecular basis of both the requirement for a previously monomethylated substrate and the lack of activity observed with di-methylated substrate. Significantly, in the crystal structure, the electron density for the co-crystallized H4K20me2 peptide was well-defined, allowing the unambiguous positioning of both di-ammonium methyl groups (Figure 2A). One of these, the ‘product’ methyl, adopts a position pointing towards the SAH sulfonium ion and represents the situation directly following methyl transfer, and the environment of the second ‘substrate’ methyl is critical to determining the specificity and is described later in the text.

In previously characterized SET domain methyltransferases, the restriction to monomethylation is determined by the presence of two tyrosine residues that interact with the substrate lysine Nε (22,23,30), shown schematically in Figure 2B. This has been termed the phenylalanine-tyrosine switch because in enzymes with a phenylalanine rather than tyrosine, the hydroxyl/water hydrogen bond network is lost, and multiple methylation states can be accommodated. Unusually, in the Suv4-20h2 ternary complex structure, a broad hydrophobic pocket accommodates the substrate methyl group whilst a direct hydrogen bond to the target lysine Nε is made by the side chain hydroxyl of Ser161 (Figure 2C). Accommodating a third methyl group would require breaking this hydrogen bond and a significant rearrangement of the active site. This would be energetically unfavourable and explains why we do not observe significant activity with an H4K20me2 substrate (Figure 2D). Mutation of Ser161 to alanine resulted in an enzyme not only with reduced overall activity with respect to the wild-type but interestingly the specificity profile was also altered. Although the Suv4-20h2(Ser161Ala) enzyme did not methylate either an unmodified or monomethylated H4K20 substrate, unlike the wild-type, it methylates a previously di-methylated peptide (Figure 2D). We attribute the loss of activity with the mono-methylated substrate to the inability to effectively restrain the lysine Nε in the optimal position for methyl transfer due to loss of the Ser161 hydroxyl hydrogen bond. We propose that the significant activity observed for the combination of the alanine mutation and histone H4K20me2 substrate is likely to arise from better restraint of the more bulky di-methyl ammonium group in the active site (Figure 2E).

But what of the requirement for monomethylated substrate? The side-chain of Phe191 occupies the position corresponding to the tyrosine-phenylalanine switch residue described for other SET domain proteins (31). A detailed comparison of the PR-Set7 and Suv4-20h2 binding sites is shown in Supplementary Figure S1A. The Suv4-20h2 Phe191 residue is located on a different region of the protein chain than in other characterized proteins (Figures 1A and 2A). This residue forms the centre of the broad hydrophobic pocket that also comprises the side chains of Trp174, Ile181 and Ala178. In contrast to the canonical hydrogen bond network, this environment effectively excludes water, and therefore favours the positioning of the methyl group of the mono-methylated lysine substrate. For the SET domain to promote efficient methyl transfer, it is essential that the target lysine-Nε is optimally positioned. Not only does the Suv4-20h2 pocket accommodate the substrate methyl but also, as the methyl to Phe Cζ distance is only 3.3 Å, it is likely that this interaction constitutes a CH3-π hydrogen bond, and this effectively locks the lysine-Nε in position. Such hydrogen bonds have been described in a number of systems (41). For a non-methylated lysine in the Suv4-20h2 active site, this interaction does not take place, and as a result the lysine Nε is not fixed in the optimal position for methyl transfer, making the reaction inefficient (Figure 2D). In other enzymes, whether a Phe or Tyr is in this position is key to product specificity; we were therefore interested in the effect of substitution of this residue with a Tyr. Unfortunately, the Suv4-20h2(Phe191Tyr) mutation was unstable, but we were able to generate the equivalent mutation in Suv4-20h1. Although stable, the effect of adding the hydroxyl was a dramatic loss of activity (Supplementary Figure S1B). For Suv4-20 enzymes rather than facilitating processivity, the phenylalanine side chain is part of a pocket that ‘locks’ the substrate methyl in position and along with Ser161 defines product specificity. Analysis of the sequences of all predicted human SET domain proteins reveals that contrary to expectation most do not contain aromatic switch residues in the conventional positions (Supplementary Figure S2). This implies that a non-canonical configuration of the active site may be more frequent than the currently available subset of characterized proteins suggests.

### Do Suv4-20h1 and Suv4-20h2 differ biochemically?

**Figure 3.**
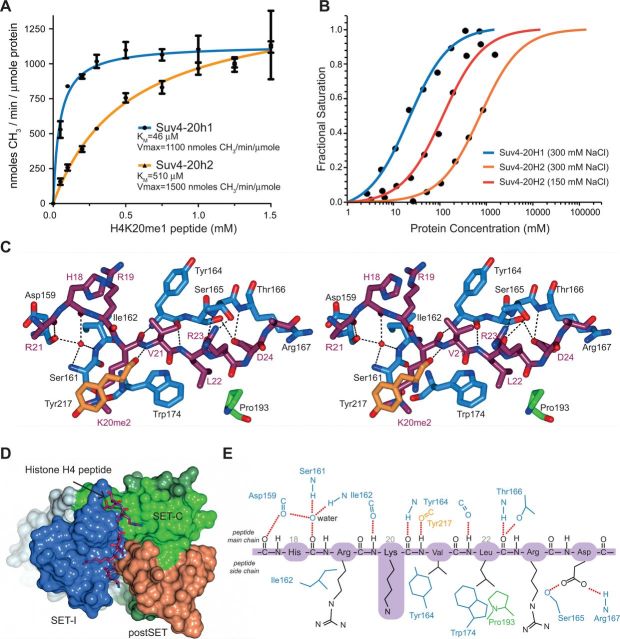
Histone H4 recognition in Suv4-20h1 and Suv4-20h2. (**A**) Methyltransferase activity Suv4-20h1 and Suv4-20h2 with an H4K20me1 peptide. (**B**) Binding affinity of Suv4-20h1 (blue) and Suv4-20h2 (red/orange) for histone H4K20me1 peptide using MST. (**C**) Stereo view showing a stick representation of the interactions between of the H4 peptide and Suv4-20h2. (**D**) Surface representation of Suv4-20h2 showing the binding of the H4 peptide. (**E**) Schematic of interactions with the H4 peptide and residues in Suv4-20h2.

Contrary to expectation, given their divergent biological roles, we found that both Suv4-20h1 and Suv4-20h2 share the same intrinsic substrate and product specificity profile (Figure 1B), but what of their other biochemical properties? Comparing the steady state enzyme kinetics of Suv4-20h1 (61-327) and Suv4-20h2 (1-168) in excess SAM with respect to peptide concentration revealed a significant difference (Figure 3A). The estimated turnover rates, derived from Vmax were similar—1100 and 1500 nmoles CH3/min/µmole protein, respectively—corresponding to a *K*_cat_ of 1.0–1.5 min^−^^1^. This is in a similar range to typical rates reported for other SET domain enzymes in *in vitro* assays with peptide substrates (31,42). However, the *K*_M_ values with respect to monomethylated peptide substrate varied substantially, 46 ± 1 µM for Suv4-20h1 (61-327) and 510 ± 1 µM Suv4-20h2 (1-268), respectively. This suggests significantly different substrate affinity, and to quantify this, we used MST to measure the binding affinity for a fluorescein-tagged histone H4K20me1 peptide (Figure 3B). The Suv4-20h1 (61-327) SET domain construct binds the peptide with a dissociation constant (*K*_d_) of 21 ± 1 μM, in a buffer containing 300 mM NaCl. In the equivalent buffer, the Suv4-20h2 (1-268) construct binding is extremely weak (>700 μM) and difficult to evaluate, but in a buffer containing only 150 mM NaCl, the estimated *K*_d_ of Suv4-20h2 for peptide was 114 ± 5 μM. Unfortunately, the recombinant Suv4-20h1 (61-327) construct was not stable in the lower salt buffer. The sensitivity of peptide substrate binding of the Suv4-20h2 (1-268) to the buffer ionic strength is also reflected in its methyltransferase activity (Supplementary Figure S1C).

### Recognition of Histone H4 by Suv4-20h2

The molecular basis of the differential substrate affinity of Suv4-20h1 and Suv4-20h2 is not readily apparent, as the residues that interact with the substrate are well conserved (Figure 1A). The histone H4 peptide binds in an extended conformation to a groove formed at the interface of the Suv4-20h2 SET-I domain with the postSET and SET-C region (Figure 3D). The H4 residues (17–24 of histone H4) pack in a parallel orientation alongside the SET-I domain strand (residues 162–166) with which it forms a series of polar and hydrophobic interactions (Figure 3C). Surprisingly, given the high sequence specificity of the enzyme, the majority of polar interactions between H4 peptide and Suv4-20h2 are between main-chain atoms (shown schematically in Figure 3E). These include hydrogen bonds between the Lys20, Val21 and Leu22 amides to the carbonyls of Ile162 and Tyr164 in the SET-I region and Tyr217 from SET-C. A water molecule bridges interactions between the His18 carbonyl and the main-chain of Asp169, Ser161 and Ile162. With the exception of Asp24, the peptide side-chain contacts with the protein are hydrophobic. This is notable different to the basis of recognition of histone H4 by PR-Set7, where the Arg17, His18, Arg19 and Arg23 side-chains all make hydrogen bonds with the protein (23). Indeed, a detailed analysis of residue preference at each position revealed that residues Lys17 and His18 were key to recognition (43). In PR-Set7, the histone peptide His18 residue was shown to be critical for recognition, as it completes the lysine-binding channel and is required for activity (22,44), but in Suv4-20h2, the H4 His18 is orientated away from the sprotein core and towards the solvent. Thus, the determinants of histone H4 sequence specificity are not conserved between PR-Set7 and the Suv4-20 family.

### Cofactor binding

**Figure 4.**
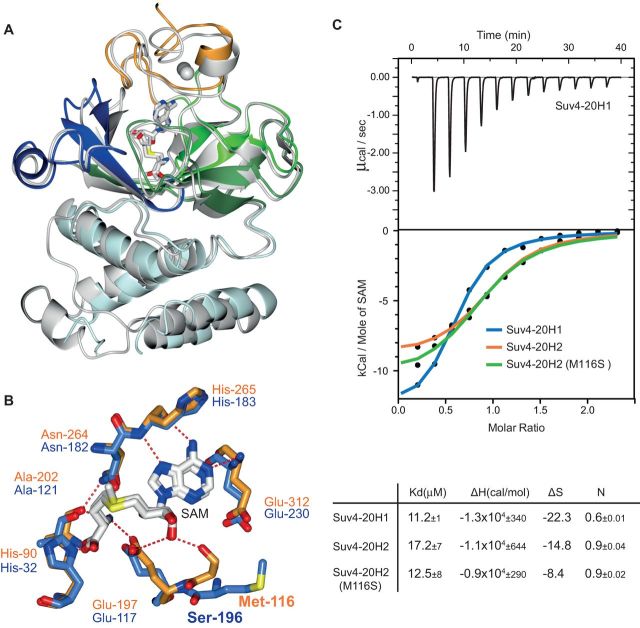
Analysis of cofactor recognition in Suv4-20 enzymes. (**A**) Cartoon representation of the structure of the binary complex of mouse Suv4-20h1 (61-327) with SAM superposed on the Suv4-20h2 structure. The Suv4-20h1 is coloured as in Figure 1C and Suv4-20h2 in grey. A stereo view of this interaction is shown in Supplementary Figure S4A. (**B**) Overlay of a stick representation of the cofactor binding interactions observed in mouse Suv4-20h1 binary complex with SAM and Suv4-20H2 ternary complex with SAH. A stereo view of this interaction is shown in Supplementary Figure S4B. (**C**) Determination of SAM binding affinity for Suv4-20h1, Suv4-20h2 and Suv4-20h2(M116S) mutant by isothermal calorimetry. The binding curves for all three proteins are overlayed for simplicity, and the original data are presented in full in Supplementary Figure S4C. Binding constants, enthalpy and entropy measurements are summarized below the graph.

In addition to the product complex of Suv4-20h2 with SAH and peptide, we have determined the structure of a binary complex of mouse Suv4-20h1 (61-327) paralog with SAM, at a resolution of 2.2 Å. The structural conservation between the two proteins in the region covered by the constructs is very high, both over the SET domain and the alpha helical N-flanking region (Figure 4A). Comparing the environment of cofactor binding in the complexes reveals that the majority of these interactions are also conserved (Figure 4B). However, Suv4-20h1 has an additional hydrogen bond between residue Ser196 and the SAM carbonyl O3′, the equivalent residue in Suv4-20h2 is a methionine. The consequence of this additional bond is only a modest increase in affinity for SAM. Using isothermal calorimetry, in a buffer containing 500 mM NaCl to stabilize the protein, we determined the binding constants (*K*_D_) for mouse Suv4-20h1 (61-327) and Suv4-20h2 (1-268) to be 11.2 ± 1 µM and 17.2 ± 8 µM, respectively (Figure 4C). Mutating the Suv4-20h2 methionine to serine resulted in a dissociation constant of 12.5 ± 7 µM, (Figure 4C and Supplementary Figure S4). Given the narrow range of binding constants, it is unlikely that SAM binding represents a physiologically significant difference between the paralogs.

## DISCUSSION

An epigenetic signalling network based on the post-translational modification of histones requires that both the modification machinery that generate marks and the recognition domains on the effector proteins recruited to these marks exhibit a high degree of specificity. The Suv4-20 enzyme family has a distinct specificity profile, namely, the requirement for a histone H4 previously monomethylated on lysine-20 and the production of an H4K20me2 product. Analysis of the structure of the Suv4-20h2 product complex reveals that a novel mechanism generates specificity. The substrate methyl group is recognized by a hydrophobic pocket, and further methylation is prevented by a tight hydrogen bond between Ser161 and the substrate lysine Nε. This represents the first description of a SET domain protein in which a limitation to processivity is achieved by a mechanism other than the canonical phenylalanine-tyrosine switch. However, sequence analysis indicates that characterization of further predicted SET domain proteins may reveal a variety of mechanisms to achieve methyl transfer specificity.

As PR-Set7 is limited to production of H4K20me1, it was proposed that a sequential mechanism exists to produce the H4K20 methyl marks on nucleosomes in higher organisms (16). Our structural analysis and supporting biochemical data indicate that the Suv4-20 enzymes add the next methyl to produce the H4K20me2 mark. However the *in vitro* specificity profile and the configuration of the active site are not consistent with H4K20me3 production. This implies that a third enzyme, or enzyme family, may be responsible for the H4K20me3 mark identified in facultative heterochromatin and pericentric chromatin. We cannot rule out that a shift in specificity *in vivo* might be induced by a hitherto unknown mechanism involving the C-terminus of the protein, recruitment by HP1 or the nucleosomal substrate. All three methylation states have been attributed to the related Set9 protein in *S.pombe* (5), which may indicate flexibility in the active site in this ortholog. In *Saccharomyces cerevisiae*, which had previously been thought to lack histone H4 methylation and lacks a Suv4-20 homolog, the H4K20me1 mark has now been identified, and this may point to an as yet unidentified H4K20 monomethylase family in yeast (45). In mammals where the sequential addition of H4K20 is more established, the SMYD3 protein has recently been shown to have H4K20me2 to H4K20me3 activity (46). Furthermore, superposition of the SMYD3 structure with the Suv4-20h2 ternary complex indicates that the SMYD3 active site has a hydrophobic pocket in an analogous position to that of Suv4-20h2, which we propose could accommodate one methyl group. In the position equivalent to the Suv4-20h2 Ser161, there is an environment that may be able to accommodate the extra methyl group (described in Supplementary Figure S5). Further work is required to confirm SMYD3 involvement in H4K20 signalling, and it is worth noting that the specificity of the majority of SET domain proteins in the human genome is yet to be determined. It is possible that echoing the loss of H4K20me1, -me2 and -me3 on deletion of PR-Set7, the loss of both H4K20me2 and -3 on deletion or knockdown of Suv4-20h2 could be due to loss of the required H4K20me2 substrate for a subsequent enzyme (13,26).

The requirement for two non-redundant H4K20me2-specific enzymes in higher organisms is still unclear, although there are many other SET domain subfamilies that share specificity, e.g. the MLL family for H3K4 or G9a/GLP for H3K9 (47,48). It has been observed that Suv4-20h1 is more ubiquitously expressed, both in terms of tissue type and developmental stage, than Suv4-20h2 (26). Our data indicate that although Suv4-20h1 and Suv4-20h2 share the same product specificity profile, they do show some differences in their peptide binding, substrate binding and steady-state enzyme kinetic properties. The Suv4-20h1 steady state properties are more similar to the Suv4-20 from *Drosophila*, which like other less advanced organisms has only a single paralog (Supplementary Figure S6). Although the Suv-20 paralogs exhibit high structural conservation over the extent of the crystallographic constructs, their sequences diverges at the C-terminus and that Suv4-20h1 has an N-terminal extension. Presumably, these regions have an important role in mediating interactions and localization. For example, it has recently been reported that Suv4-20h2 associates more stably with pericentric heterochromatin than its Suv4-20h1 paralog (49). It may be that the differences we have observed in intrinsic biochemical properties reflects the challenges of the chromatin environment in which the enzymes function, but further research will determine whether other factors, such as post-translational modifications to either substrate or enzyme, or the effect of partner proteins, may have a significant bearing on activity.

## SUPPLEMENTARY DATA

Supplementary Data are available at NAR Online.

## FUNDING

The Institute of Cancer Research and benefits from infrastructural support for structural biology by Cancer Research UK. We acknowledge NHS funding to the NIHR Biomedical Research Centre. Funding for open access charge: the Medical Research Council [Unit Programme number U117584222].

*Conflict of interest statement*. None declared.

## Supplementary Material

Supplementary Data
