# Supplementary material for: A novel route to product specificity in the Suv4-20 family of histone H4K20 methyltransferases
Source: Nucleic Acids Res. 2013 Sep 18;42(1):661–71. doi: 10.1093/nar/gkt776 (PMC3874154; doi:10.1093/nar/gkt776)
Supplement: Supplementary Data [file supp_42_1_661__index.html]

A novel route to product specificity in the Suv4-20 family of histone H4K20 methyltransferases — A novel route to product specificity in the Suv4-20 family of histone H4K20 methyltransferases — Supplementary Data 

# A novel route to product specificity in the Suv4-20 family of histone H4K20 methyltransferases

## Supplementary Data

files

**Files in this Data Supplement:**

- Supplementary Data - pdf file
