# Supplementary material for: A novel route to product specificity in the Suv4-20 family of histone H4K20 methyltransferases
Source: Nucleic Acids Res. 2013 Sep 18;42(1):661–71. doi: 10.1093/nar/gkt776 (PMC3874154; doi:10.1093/nar/gkt776)

A novel route to product specificity in the Suv4-20 family of histone H4K20 methyltransferases

Stacey M. Southall, Nora B. Cronin and Jon R. Wilson

Supplementary Data

S1. Analysis of the basis of Suv-20 activity.

S2. Sequence comaprison of human SET proteins.

S3. Peptide binding using Microscale Thermophoresis

S4. Analysis of cofactor binding in Suv4-20h1 and Suv4-20h2.

S5. Origins of Suv4-20h1 and Suv4-20h2.

S6. Analysis of the SMYD3 active site.

S1. Analysis of the basis of Suv4-20 actvity.

S1A. Comparison of target lysine binding in the active sites of PR-Set7 and Suv4-20H2.

The panels on the left show views of the PR-Set7 active site from the side and from the top (from PDB: 2BQZ). The panels on the right show the equivalent views of the Suv4-20h2 active site. In place of the hydrogen bond interaction observed in PR-Set7 between the lysine Nε and the Tyr245 hydroxyl in Suv4-20h2 there is a hydrogen bond with the Ser161 hydroxyl. In PR-Set7 the second position on the lysine Nε is involved to a hydrogen bond to a water molecule which in turn makes a strong hydrogen bond to Tyr334. In Suv4-20 their are no conventional hydrogen bonds at this position, but the substrate methyl is presented to a large hydrophobic pocket, at the centre of which is the sidechain of Phe191. The distance between the substrate methyl and the Cζ atom on Phe191 is only 3.3Å and therefore this likely represents a CH-π hydrogen bond; the methyl - Phe interaction positions the lysine for optimal methyl transfer.

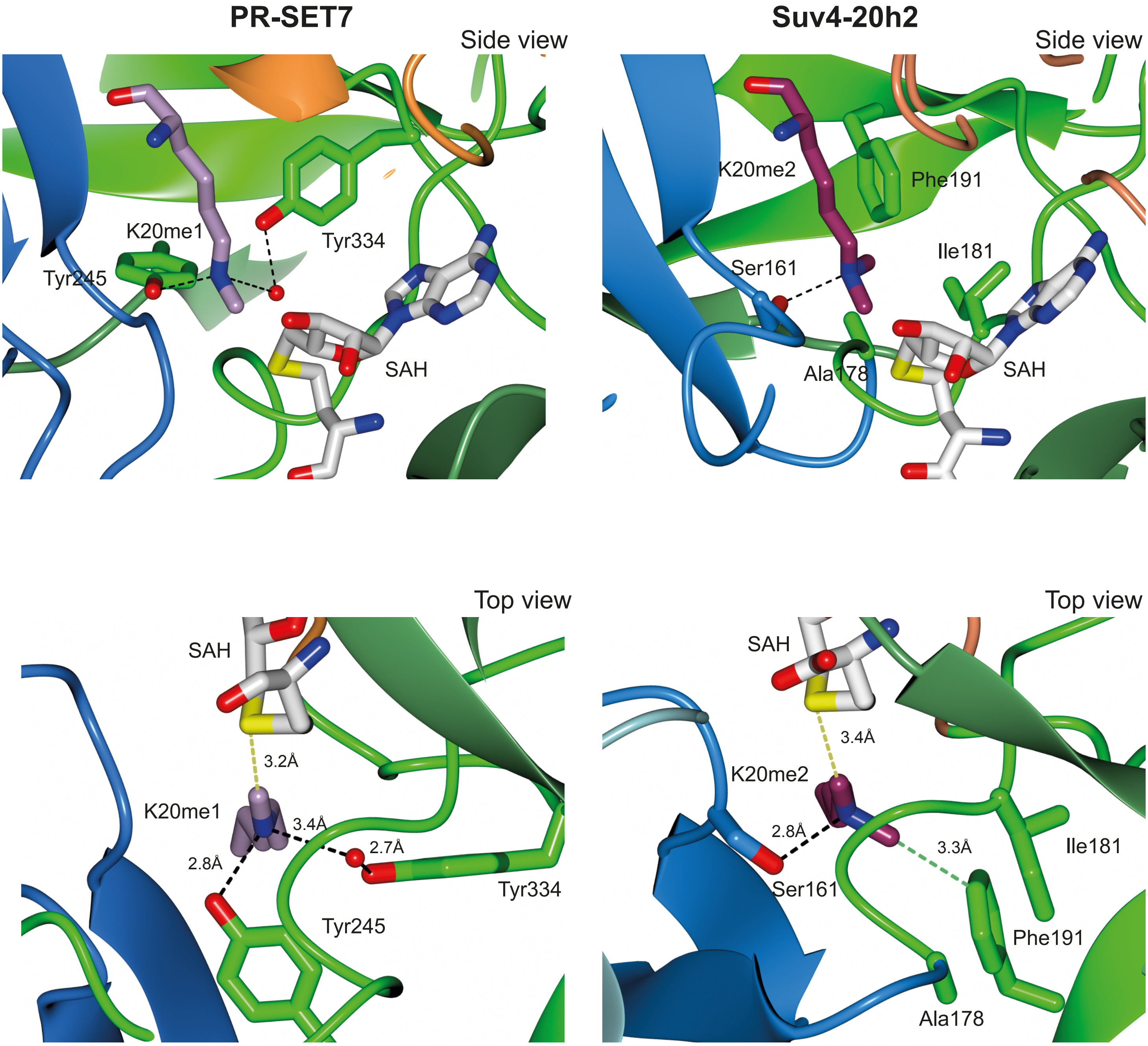

S1B. Activity of a Suv4-20h1(F237Y) mutant

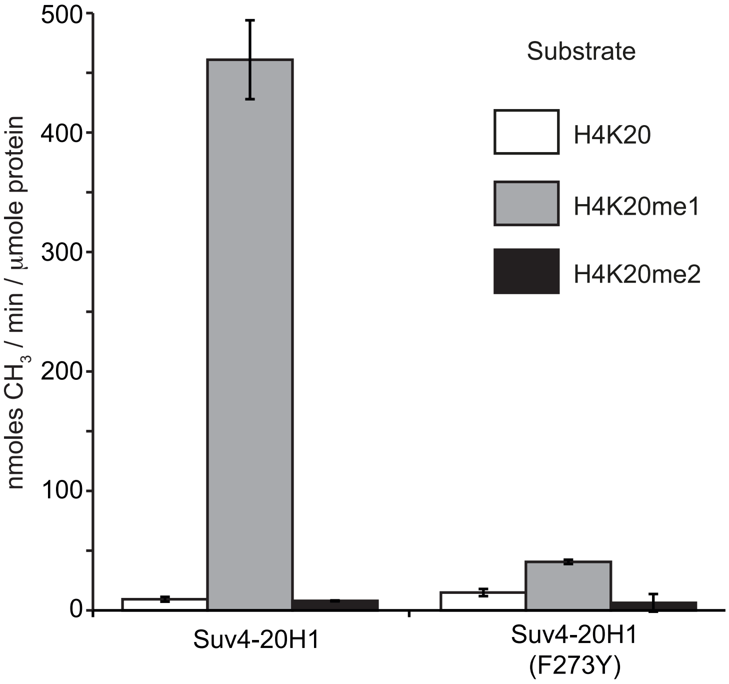

Specificity profile of a Suv4-20h1(F237Y) mutant in a methyltransferase assay with unmodified, H4K20me1 or H4K20me2 substrate. The activity of the mutant enzyme is severely reduced compared to the wild-type, presumably due to the limited space in the active site.

S1C. Salt dependence of Suv4-20h2

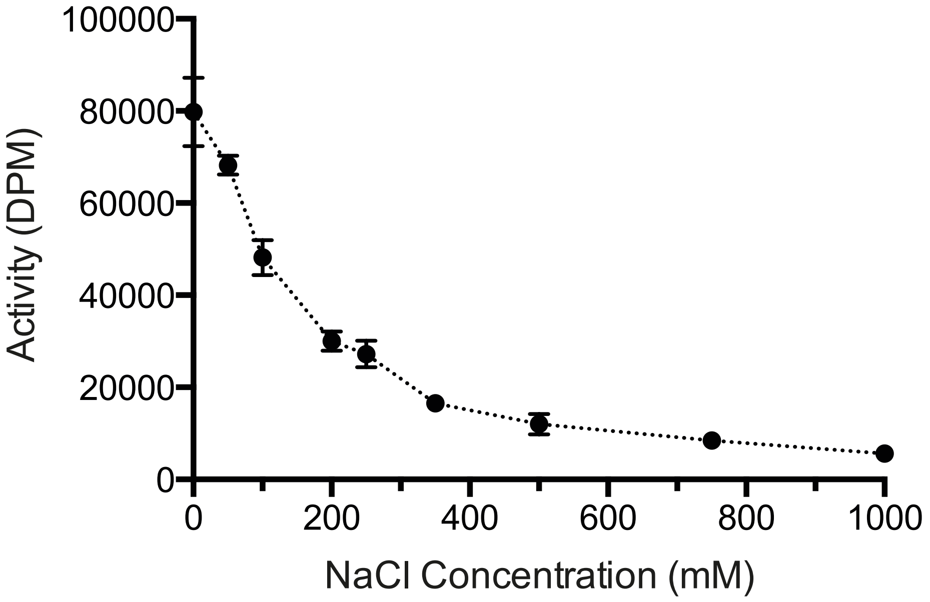

Methyltransferase activity of Suv4-20h2(1-246), with 500 μM H4K20me1 peptide, 500 μM SAM in reaction buffer containing 50 mM Tris, pH8.5, 1 mM DTT and increasing concentrations of NaCl. The enzyme shows a very clear dependence on the salt concentration of the reaction buffer.

S2. Sequence comparison of human SET domain proteins

Multiple sequence alignment of the SET domain proteins in the human genome reveals that the occurrence of Phe or Tyr residues at the canonical positions in the SET-N region (A) or SET-C region (B) is less conserved than would be expected from analysis of the currently well-characterised subset of proteins. At the B position there is significant variance and this suggests that like the Suv4-20 family many SET domain family proteins may not control specificity through the Phe-Tyr switch but have evolved an alternative mechanism.

```
                 SET-N (A)                               SET-C (B)
Suv4-20H2        CTRYSMETN----GAKIVSTRAWKKNEKLELLVGCIAE   PAAFINHDCKPNCKFVPSDGN-------TACVKVLRDIEPGDEVTCFYGEG
SUV4-20H1        CNRYSSEQN----GAKIVATKEWKRNDKIELLVGCIAE   PAAFINHDCRPNCKFVSTGRD-------TACVKALRDIEPGEEISCYYGDG
PR-Set7          GMKIDLIDG---KGRGVIATKQFSRGDFVVEYHGDLIE   LGRLINHSKCGNCQTKLHDIDGV----PHLILIASRDIAAGEELLYDYGDR
SET7/9           AESLISSAG-----EGLFSKVAVGPNTVMSFYNGVRIT   LGHKANHSFTPNCIYDMFVHPRF---GPIKCIRTLRAVEADEELTVAYGYD
NSD1             V-EIFRTLQ---RGWGLRTKTDIKKGEFVNEYVGELID   YARFMNHCCQPNCETQKWSVNGD----TRVGLFALSDIKAGTELTFNYNLE
NSD2             T-KIIKTDG---KGWGLVAKRDIRKGEFVNEYVGELID   YSRFMNHSCQPNCETLKWTVNGD----TRVGLFAVCDIPAGTELTFNYNLD
NSD3             A-EIIKTER---RGWGLRTKRSIKKGEFVNEYVGELID   YSRFMNHSCNPNCETQKWTVNGD----VRVGLFALCDIPAGMELTFNYNLD
ASH1L            CLERFRAEE---KGWGIRTKEPLKAGQFIIEYLGEVVS   EARFINHSCDPNCEMQKWSVNGV----YRIGLYALKDMPAGTELTYDYNFH
MLL1             AVGVYRSPI---HGRGLFCKRNIDAGEMVIEYAGNVIR   AARFINHSCEPNCYSRVINIDGQ----KHIVIFAMRKIYRGEELTYDYKFP
MLL2             NVYLARSRI---QGLGLYAAKDLEKHTMVIEYIGTIIR   PARYINHSCAPNCVAEVVTFDKE----DKIIIISSRRIPKGEELTYDYQFD
MLL3             NVYLARSRI---QGLGLYAARDIEKHTMVIEYIGTIIR   PARYINHSCAPNCVAEVVTFERG----HKIIISSSRRIQKGEELCYDYKFD
MLL4             AVGVYRSAI---HGRGLFCKRNIDAGEMVIEYSGIVIR   AARFINHSCEPNCFSRVIHVEGQ----KHIVIFALRRILRGEELTYDYKFP
MLL5             KPPVESHIQK--NKKILKSAKDLPPDALIIEYRGKFML   EARFIRRSCTPNAEVRHEIQDGT----IHLYIYSIHSIPKGTEITIAFDFD
G9a              VRLQLYRTAK--MGWGVRALQTIPQGTFICEYVGELIS   ISRFINHLCDPNIIPVRVFMLHQDLRFPRIAFFSSRDIRTGEELGFDYGDR
GLP              ARLQLYRTRD--MGWGVRSLQDIPPGTFVCEYVGELIS   VSRFINHHCEPNLVPVRVFMAHQDLRFPRIAFFSTRLIEAGEQLGFDYGER
SUV39H1          DLCIFRTDDG--RGWGVRTLEKIRKNSFVMEYVGEIIT   ISHFVNHSCDPNLQVYNVFIDNLDERLPRIAFFATRTIRAGEELTFDYNMQ
SUV39H2          SLCIFRTSNG--RGWGVKTLVKIKRMSFVMEYVGEVIT   VSHFVNHSCDPNLQVFNVFIDNLDTRLPRIALFSTRTINAGEELTFDYQMK
SETMAR           HFQVFKTHK---KGWGLRTLEFIPKGRFVCEYAGEVLG   IGRFLNHSCEPNLLMIPVRIDS---MVPKLALFAAKDIVPEEELSYDYSGR
SMYD1            FLQYWPPQS------QQFSMQYISHIFGVINCNGFTLS   TVIFNNGNHEAVKSMFHTQMR--------IELRALGKISEGEELTVSYIDF
SMYD2            VKEFESHLD-----KLDNEKKDLIQSDIAALHHFYSKH   DVALMNHSCCPNVIVTYK--------GTLAEVRAVQEIKPGEEVFTSYIDL
SMYD3            FYDLESNIN-----KLTEDKKEGLRQLVMTFQHFMREE   SISLLNHSCDPNCSIVFN--------GPHLLLRAVRDIEVGEELTICYLDM
SMYD4            NSSQLKAAVTP----ELCPDVTIWGVAMLRHMLQLQCN   VISLLNHSCSPNTSVSFISTVAT--------IRASQRIRKGQEILHCYGPH
SMYD5            VKQAKDKDRWIRLFSQFCNKTANEEEEIVHKLLGDKFK   TSSLSQWVHACDTLELKPQDREQ---LDAFIDQLYKDIEAATGEFLNCEGS
WHSC1            T-KIIKTDG---KGWGLVAKRDIRKGEFVNEYVGELID   YSRFMNHSCQPNCETLKWTVNGD----TRVGLFAVCDIPAGTELTFNYNLD
PRDM1            YATNSEE------VIGVMSKEYIPKGTRFGPLIGEIY-   WMRYVNPAHSPREQNLAACQN-----GMNIYFYTIKPIPANQELLVWY---
PRDM4            RQSIVGAEVG------VWTGETIPVRTCFGPLIGQQSH   WMMFVRKARNREEQNLVAYPH-----DGKIFFCTSQDIPPENELLFYYSRD
PRDM5            LKSSRVQD-----GMGLYTARRVRKGEKFGPFAGEKRM   WLRFVHEAPSQEQKNLAAIQE-----GENIFYLAVEDIETDTELLIGYLDS
PRDM6            CTSTVPGL-----AYGICAAQRIQQGTWIGPFQGVLLP   WMRYIRCARHCGEQNLTVVQY-----RSNIFYRACIDIPRGTELLVWYNDS
PRDM7            GPSGIPQAGLG----VWNEASDLPLGLHFGPYEGRIT-   WMRYVNCARDDEEQNLVAFQY-----HRQIFYRTCRVIRPGCELLVWSGDE
PRDM8            GDAKAVQQCLTDIFTSVYTTCDIPENAIFGPCVLSHTS   WLRLVQSARDKEEQNLEAYIKNG-----QLFYRSLRRIAKDEELLVWYGKE
PRDM9            GPSGIPQAGLG----VWNEASDLPLGLHFGPYEGRIT-   WMRYVNCARDDEEQNLVAFQY-----HRQIFYRTCRVIRPGCELLVWYGDE
SETD1A           KLRFGRSRI---HEWGLFAMEPIAADEMVIEYVGQNIR   LARFINHCCTPNCYAKVITIESQ----KKIVIYSKQPIGVDEEITYDYKFP
SETD2            V-EVILTEK---KGWGLRAAKDLPSNTFVLEYCGEVLD   CSRFMNHSCEPNCETQKWTVNGQ----LRVGFFTTKLVPSGSELTFDYQFQ
SETD3            TRQNQIPTEDGSRVTLALIPLWDMCNHTNGLITTGYNL   AEVLARAGIPTSSVFALHFTEPPI--SAQLLAFLRVFCMTEEELKEHLLGD
SETD6A           SQHTCSIGG-----LLERERVALQSQSGWVPLLLALLH   ADI LNHLANHNANLEYSAN--------CLRMVATQPIPKGHEIFNTYGQM
SETD6B           SQHTCSIGG-----LLERERVALQSQSGWVPLLLALLH   AADILNHLANHNANLEYSAN--------CLRMVATQPIPKGHEIFNTYGQM
SETD7            AESLISSAG-----EGLFSKVAVGPNTVMSFYNGVRIT   LGHKANHSFTPNCIYDMFVHPRFGPI---KCIRTLRAVEADEELTVAYGYD
SETD8            GMKIDLIDG---KGRGVIATKQFSRGDFVVEYHGDLIE   LGRLINHSKCGNCQTKLHDIDGV----PHLILIASRDIAAGEELLYDYGDR
E(Z)like         T-KLFKTEG---RGWGLLADEDIKAGQFVIEYCGEVIS   LARFINHSCQPNCETRKWNVLGE----IRVGIFAKHDIPIGTELAYDYNFE
MG44             TSASSARMMG--VAGASAPWRRFARTASSWSTWERSLP   ISHFVNHSCDPNLQVYNVFIDNLDERLPASLSLPQEPSGQARSSPLITTCK
EVI1             RESNMPGAG-----LGIWTKRKIEVGEKFGPYVGEQRS   WLKYIRFAGCYDQHNLVACQI-----NDQIFYRVVADIAPGEELLLFMKSE
SETB1            RLQLFKTQN---KGWGIRCLDDIAKGSFVCIYAGKILT   GTEDPEESNDDSSDDNFCKDEDFSTSSVWRSYATRRQTRGQKENGLSETTS
SETB2            RLQVFKTEQ---KGWGVRCLDDIDRGTFVCIYSGRLLS   TAKTEKCPPKFSNNPKELTVETKYDNISRIQYHSVIRDPESKTAIFQHNGK
PRD2             FPSAVDKT-----RIGVWATKPILKGKKFGPFVGDKK-   WLRYVNWACSGEEQNLFPLEI-----NRAIYYKTLKPIAPGEELLVWYNGE
PRD11            VKDTSGESDVR----CVNEV--IPKGHIFGPYEGQIS-   WMRYVVISREEREQNLLAFQH-----SERIYFRACRDIRPGEWLRVWYSED
PRD12            AQSSIPGE-----GLGIFSKTWIKAGTEMGPFTGRVIA   WMTYIKCARNEQEQNLEVVQI-----GTSIFYKAIEMIPPDQELLVWYGNS
PRD13            AVGVYRSAI---HGRGLFCKRNIDAGEMVIEYSGIVIR   AARFINHSCEPNCFSRVIHVEGQ----KHIVIFALRRILRGEELTYDYKFP
PRD14            PDSSGSDSLPQVPHFGVFCSSFIAKGVRFGPFQGKVV-   WMSYVNCARFPKEQNLVAVQC-----QGHIFYESCKEIHQNQELLVWYGDC
PRD15            RRLEDGAEG-------VFAITQLVKRTQFGPFESRR--   WMMLVRPAAEAEHQNLTAYQH-----GSDVYFTTSRDIPPGTELRVWYAAF
PRD15isoform2    RRLEDGAEG-------VFAITQLVKRTQFGPFESRR--   WMMLVRPAAEAEHQNLTAYQH-----GSDVYFTTSRDIPPGTELRVWYAAF
PRD16            RESSIPGAG-----LGVWAKRKMEAGERLGPCVVVPRA   WLKYIRVACSCDDQNLTMCQI-----SEQIYYKVIKDIEPGEELLVHVKEG
ENSP00000264646  HLLLAPSDVAG---WGTFIKESVQKNEFISEYCGELIS   KIRFANHSVNPNCYAKVVMVNGD----HRIGIFAKRAIQAGEELFFDYRYS
ENSP00000383605  FPSAVDKT-----RIGVWATKPILKGKKFGPFVGDKK-   WLRYVNWACSGEEQNLFPLEI-----NRAIYYKTLKPIAPGEELLVWYNGE
UPI000198CFFD    NVYLARSRI---QGLGLYAARDIEKHTMVIEYIGTIIR   PARYINHSCAPNCVAEVVTFERG----HKIIISSSRRIQKGEELCYDYKFD
ENSP00000301067  NVYLARSRI---QGLGLYAAKDLEKHTMVIEYIGTIIR   PARYINHSCAPNCVAEVVTFDKE----DKIIIISSRRIPKGEELTYDYQFD
Q59FW0_CLLL8     RLQVFKTEQ---KGWGVRCLDDIDRGTFVCIYSGRLLS   TAKTEKCPPKFSNNPKELTVETKYDNISRIQYHSVIRDPESKTAIFQHNGK
ENSP00000403000  HFQVFKTHK---KGWGLRTLEFIPKGRFVCEYAGEVLG   IGRFLNHSCEPNLLMIPVRIDS---MVPKLALFAAKDIVPEEELSYDYSGR
B0UZY0           RLQLYRTAK---MGWGVRALQTIPQGTFICEYVGELIS   ISRFINHLCDPNIIPVRVFMLHQDLRFPRIAFFSSRDIRTGEELGFDYGDR
Q8IZU7           GLDTSPG----------WVVR-----------------   WLKYIRFAGCYDQHNLVACQI-----NDQIFYRVVADIAPGEELLLFMKSE
AE6B4C           KLKFCKSHI---HDWGLFAMEPIAADEMVIEYVGQNIR   FARFINHSCNPNCYAKVITVESQ----KKIVIYSKQHINVNEEITYDYKFP
ENSP00000351046  -LCDYK--------------------------VVKVIT   VSHFVNHSCDPNLQVFNVFIDNLDTRLPRIALFSTRTINAGEELTFDYQMK
ENSP00000396943  RRLEDGAEG-------VFAITQLVKRTQFGPFESRR--   WMMLVRPAAEAEHQNLTAYQH-----GSDVYFTTSRDIPPGbELRVWYAAF
UPI000198C843    RRLEDGAEG-------VFAITQLVKRTQFGPFESRR--   WMMLVRPAAEAEHQNLTAYQH-----GSDVYFTTSRDIPPGTELRVWYAAF
euchrKMT1        RLQLYRTRD---MGWGVRSLQDIPPGTFVCEYVGELIS   VSRFINHHCEPNLVPVRVFMAHQDLRFPRIAFFSTRLIEAGEQLGFDYGER
ENSP00000384114  QLGRVTRVQK--HRKILRAARDLALDTLIIEYRGKVML   DARFIRRSCTPNAEVRHMIADGM----IHLCIYAVSAITKDAEVTIAFDYE
Q9NS29_HDCMC04P  KPPVESHIQK--NKKILKSAKDLPPDALIIEYRGKFML   EARFIRRSRTPNAEVRHEIQDGT----IHLYIYSIHSIPKGTEITIAFDFD
Q4W8S1_ZNF298d   RRLEDGAEG-------VFAITQLVKRTQFGPFESRR--   WMMLVRPAAEAEHQNLTAYQH-----GSDVYFTTSRDIPPGTELRVWYAAF
UPI000198C91A    --------------------------------------   DARFIRRSCTPNAEVRHMIADGM----IHLCIYAVSAITKDAEVTIAFDYE
```

S3. Measurement of binding affinity of Suv4-20 enzymes for histone H4K20me1 peptide using Microscale Thermophoresis.

Binding measurements by MST were performed using a NanoTemper Monoloth NT.115 Instrument (NanoTemper Technologies GmbH). The peptide tested was a N-terminally fluorescein tagged KRHRK(me1)VLRD (Pepceuticals). Measurements were made at 20 °C using 25% light-emitting diode power and 40% infrared-laser power with laser-on time was 30 s and laser-off time 5 s.

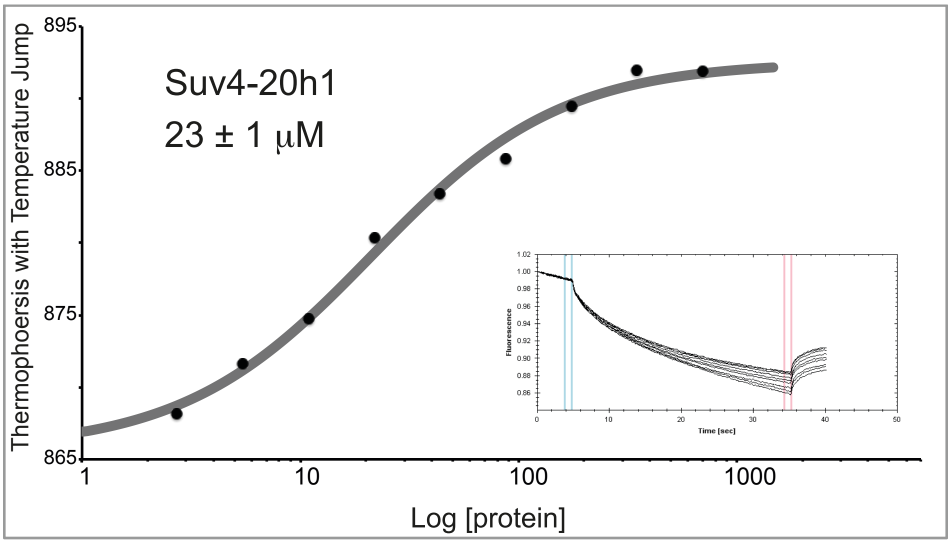

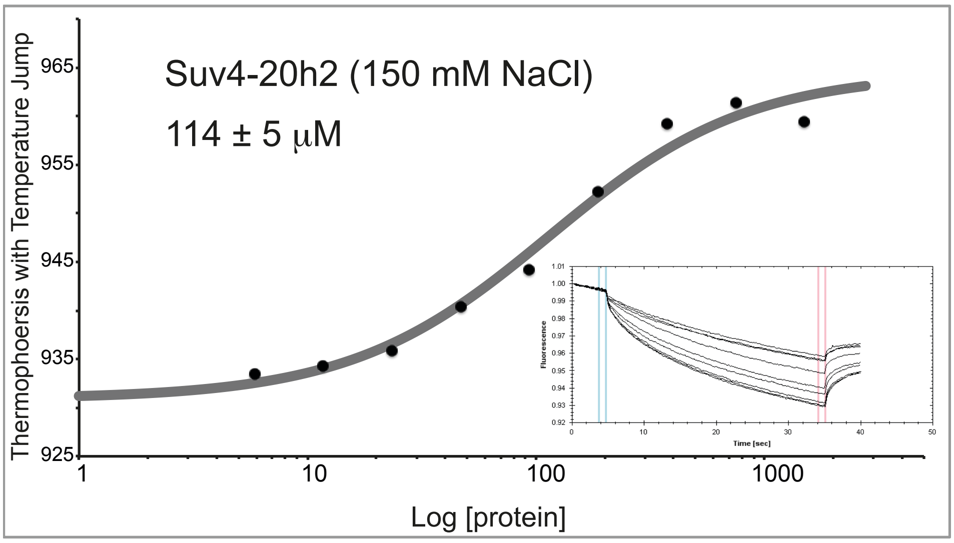

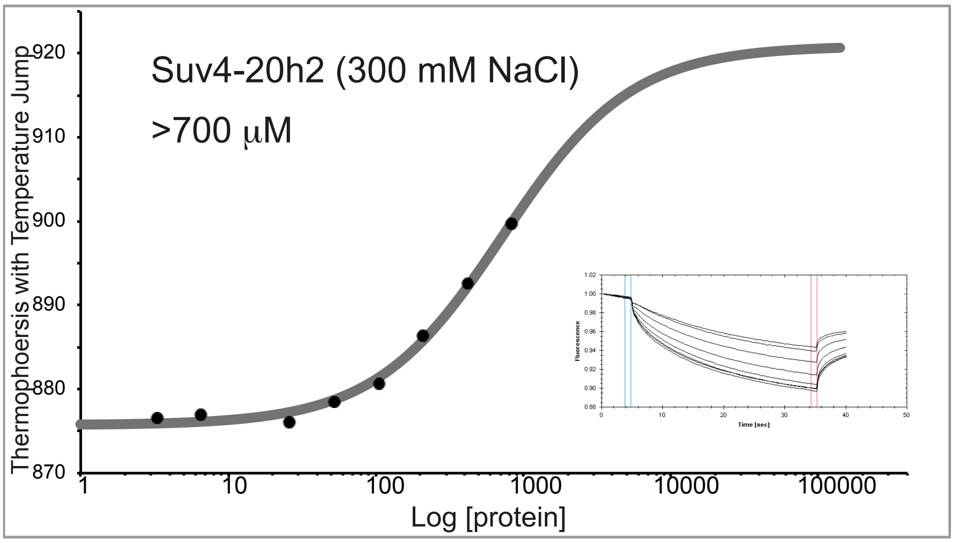

S4. Analysis of cofactor binding in Suv4-20h1 and Suv4-20h2

S4A. Stereo view of the overview of the superposition of Suv4-20h2 (coloured as per Figure 1C) and Suv4-20h1 (gray) as shown in Figure 4A.

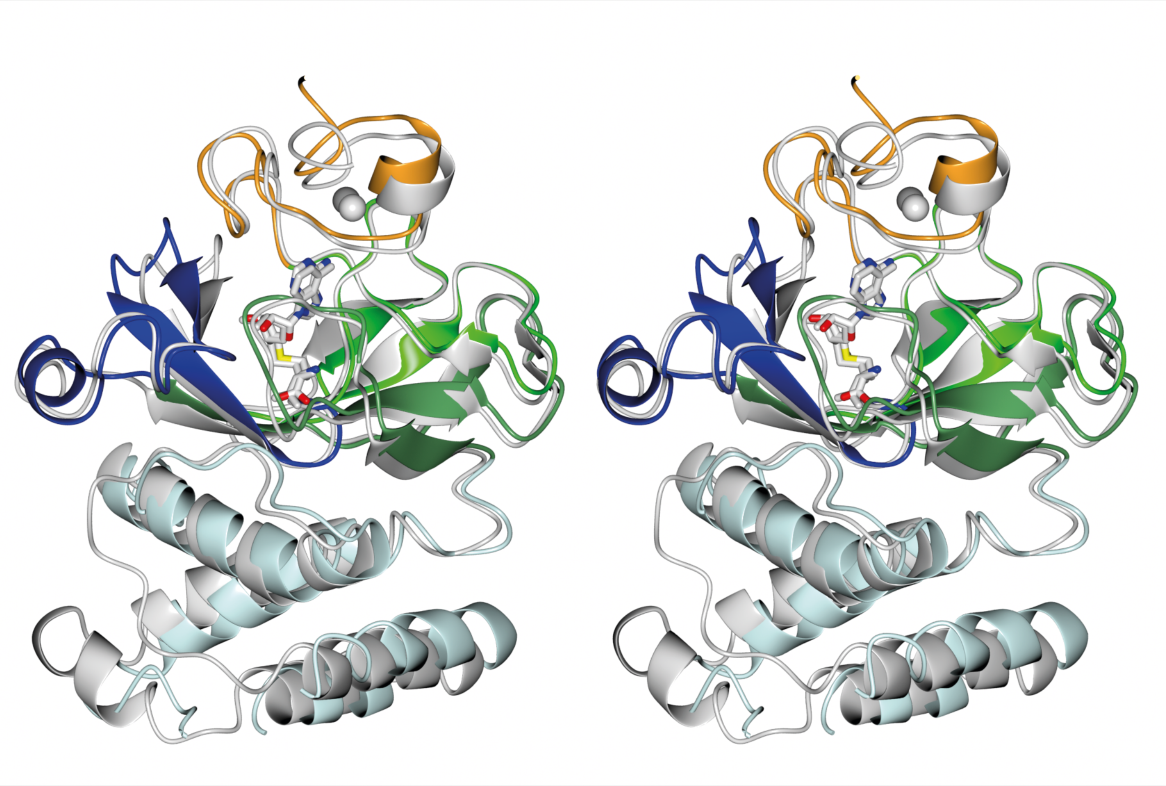

S4B. Stereo view of the overlay of the cofactor binding residues of Suv4-20h1- SAM complex (orange) and the Suv4-20h2 (blue), as shown in Figure 4B.

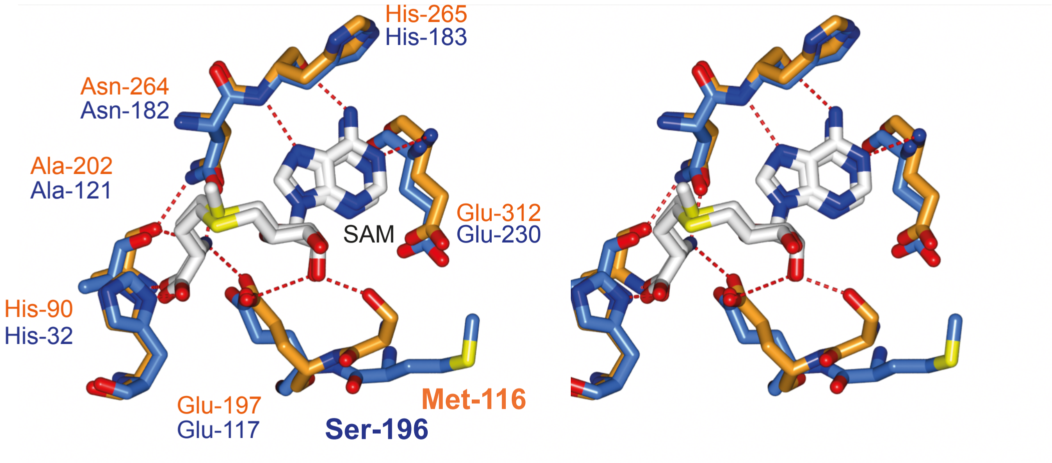

S4C. Isothermal calorimetry of SAM binding to Suv4-20 enzymes.

The Suv4-20h1 enzyme binds SAM with a moderately higher affinity than Suv4-20h2 (A. 11.2 μM compared to B. 17.2 μM). Comparing the structures of the enzymes Suv4-20h1 makes an extra interaction with SAM via Ser205 which in Suv4-20h2 is Met116. Mutation of this methionine to serine in Suv4-20h2 increases the affinity to C.12.5 μM.

A.

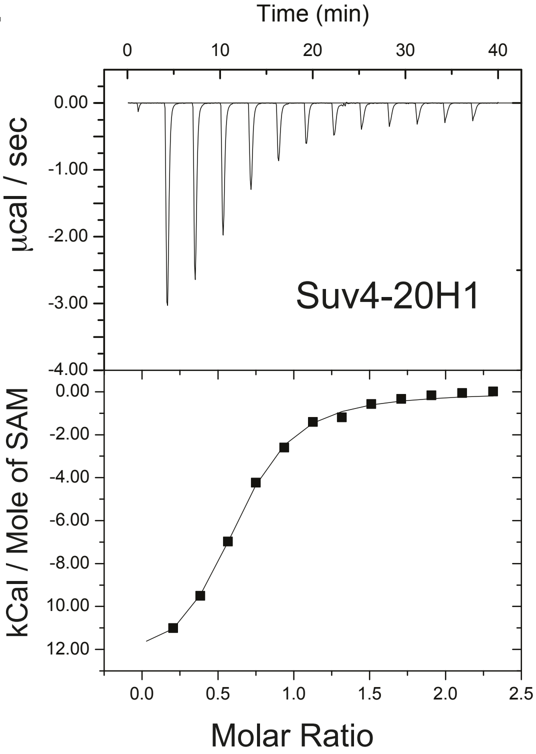

B.

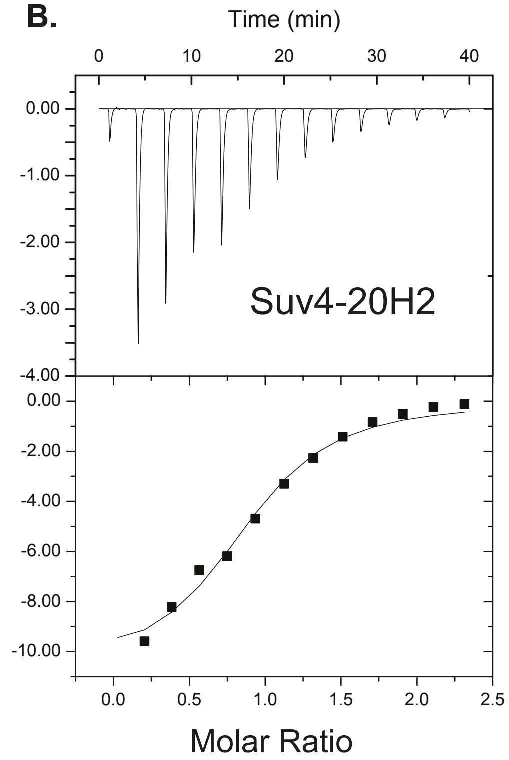

C.

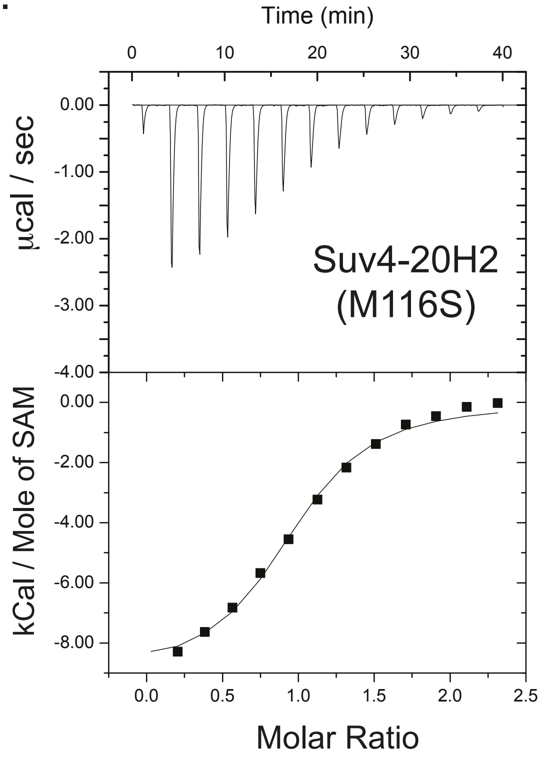

S5. Analysis of the SMYD3 active site

S5A. and **S5B**. Cartoon representations of the active site of the SMYD3 active site from the binary complex with SAH (PDB: 3OXL, Xu *et al.* (2011) Nucleic Acids Research 39, pp. 4438-4449), with equivalent colouring to that used elsewhere in the manuscript for the Suv4-20 SET domain. The gray sticks are included for reference and represent the side chain of the Ser161 and the H4K20 di-methyl lysine from the Suv4-20h2 ternary complex structure, the poisition results from the superpositioning on the respective SAH molecules. The SMYD3 acive site contains a hydrophobic pocket formed from Ile201, Ile214 and Ile237 that may accomodate a methyl group in an analogous fashion to Suv4-20h2. The arrangement of residues around the Ser161 equivalent position is somewhat more "open" and hydrophobic in character and we speculate that this may be able to accomodate a third methyl group (Shown schematically in C.). Further strcutural and biochemical analysis of SMYD3 will be required to confirm this hypothesis.

A.

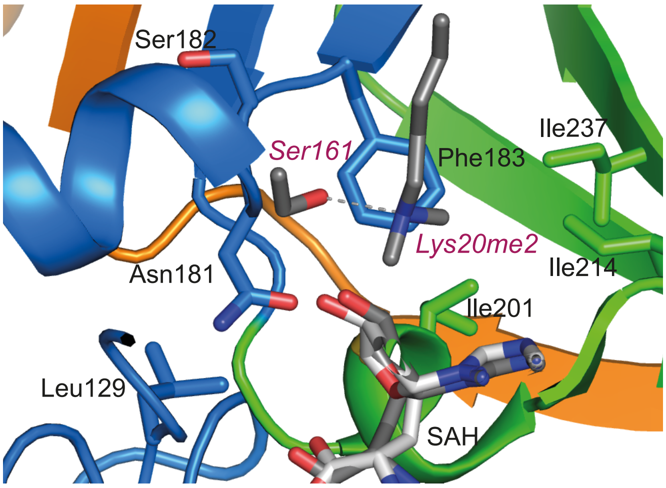

B.

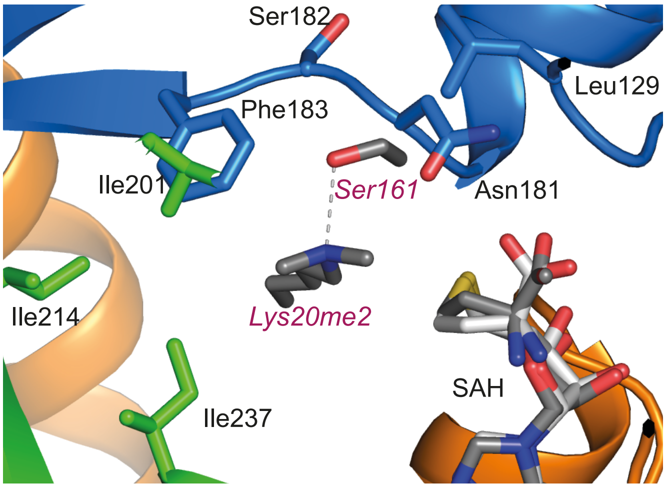

C. Schematic representation of how tri-methylation may occur in SMYD3

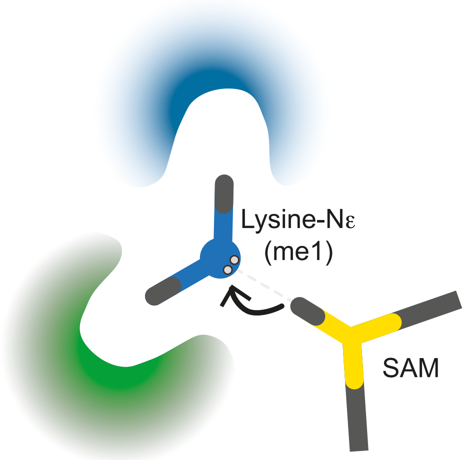

S6. Origins of Suv4-20h1 and Suv4-20h2

The comparison of the SET domain region of the Suv4-20 family of H4K20 methyltransferases across a range of species suggests that the Suv4-20h1 and Suv4-20h2 paralogs may have evolved from a common ancestor. (A) The unrooted tree from phylogenetic analysis of the SET domains from representative species creates an evolutionary context for the relationship between Suv4-20h1 and Suv4-20h2. The enzymes do not segregate together suggesting that they have evolved in parallel. (B) Methyltransferase activity of the Suv4-20 enzyme from Drosophila melanogaster with respect to H4 peptide shows similar kinetics to mouse Suv4-20h1. The Michaelis-Menten parameters were; Km=90 ±10 μM and Vmax=8001±0.2 nmol CH3/min/μmol.

A.

B.

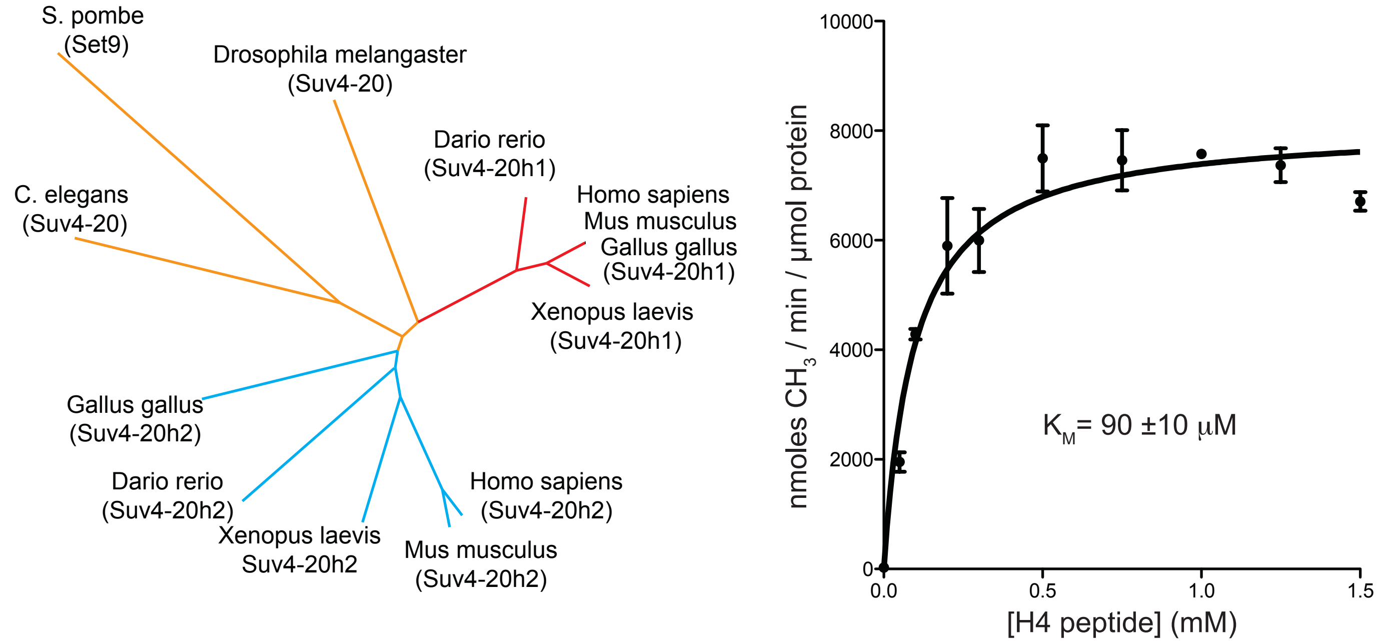

Supplement: Supplementary Data [file supp_gkt776_nar-01772-m-2013-File006.pdf]
